# A Generalized Spatial Measure for Resilience of Microbial Systems

**DOI:** 10.3389/fmicb.2016.00443

**Published:** 2016-04-07

**Authors:** Ryan S. Renslow, Stephen R. Lindemann, Hyun-Seob Song

**Affiliations:** ^1^Environmental Molecular Sciences Laboratory, Pacific Northwest National Laboratory, RichlandWA, USA; ^2^Biological Sciences Division, Earth and Biological Sciences Directorate, Pacific Northwest National Laboratory, RichlandWA, USA

**Keywords:** resilience, microbial communities, recovery, ecology, emergent properties

## Abstract

The emergent property of resilience is the ability of a system to return to an original state after a disturbance. Resilience may be used as an early warning system for significant or irreversible community transition; that is, a community with diminishing or low resilience may be close to catastrophic shift in function or an irreversible collapse. Typically, resilience is quantified using recovery time, which may be difficult or impossible to directly measure in microbial systems. A recent study in the literature showed that under certain conditions, a set of spatial-based metrics termed *recovery length*, can be correlated to recovery time, and thus may be a reasonable alternative measure of resilience. However, this spatial metric of resilience is limited to use for step-change perturbations. Building upon the concept of recovery length, we propose a more general form of the spatial metric of resilience that can be applied to any shape of perturbation profiles (for example, either sharp or smooth gradients). We termed this new spatial measure “perturbation-adjusted spatial metric of resilience” (PASMORE). We demonstrate the applicability of the proposed metric using a mathematical model of a microbial mat.

## Introduction

Complex networks of interacting components produce outcomes that cannot be easily predicted, even when the state of the network components and the inputs to the network are known. These difficult-to-determine outcomes have come to be known as emergent phenomena or higher-order properties, which emerge from the functioning of the whole network, rather than as a simple sum of the individual states of the parts (De La Fuente et al., 2008; Schubert, 2014). Emergent properties arise in complex networks that impact our lives, such as in the communities of microbes and higher organisms that compose ecosystems (Tilman et al., 2001), social networks (Lusseau, 2003; Mitrovic and Tadic, 2010), military-political structures (Porter et al., 2005), commercial systems (Carey and Carville, 2003; Cimellaro et al., 2010), and climate and weather systems (Higgins et al., 2002; Easterling and Kok, 2003). Consequently, developing the ability to understand and predict emergent properties from complex networks is of great importance.

Microorganisms are commonly found physically associated with one another in spatially structured communities such as biofilms or microbial mats (Curtis and Sloan, 2004; Wagner et al., 2006; Fuhrman, 2009; Robinson et al., 2010; Renslow et al., 2011). These communities may range from monocultures to highly diverse assemblages of species; however, in either case, individual members operate and interact in ways governed by their individual functional responses and local microenvironmental conditions (Schramm et al., 1996; Hibiya et al., 2003; Bernstein et al., 2013, 2014). Formation of a biofilm matrix composed of extracellular polymeric substances confers significant fitness advantages on the microbes it shelters, including physical protection, such as from predation or shearing forces, reduction of environmental stresses, such as from rapid changes in environmental conditions or exposure to antibiotics, facilitation of beneficial interspecies relationships, and rapid exchange of genetic material (Flemming and Wingender, 2001; Laspidou and Rittmann, 2002; Czaczyk and Myszka, 2007; Cao et al., 2011). It is likely that emergent properties arise from the spatial organization of microbes, forming microenvironments and promoting interconnectedness of a multi-species metabolic network through resource exchange and intercellular communication (Xavier and Foster, 2007; Konopka, 2009; Wintermute and Silver, 2010).

Resilience is a higher-order property in microbial communities, characterized by the ability to recover from a perturbation or disturbance (Allison and Martiny, 2008; Shade et al., 2012; Griffiths and Philippot, 2013; Hawkes and Keitt, 2015). While resilience is not yet conclusively defined in microbial communities, we use this term to imply the rate of recovery of a given *function* in a community (or, more generally, their *functional* relationship with environmental variables) after perturbation. The concept of functional resilience in both engineered and ecological microbial systems, and the relationship between state and functional properties is examined in detail in our companion paper (Song et al., 2015). Attempts at quantifying resilience have primarily been done by monitoring functional recovery over time, with resilience being negatively correlated to recovery time (i.e., the time required for function to recover a defined percent of original function), or being correlated to recovery speed (i.e., the rate of recovery of function with units of slope). Faster recovery of function is therefore an indicator of higher resilience. These ideas have been explored theoretically and experimentally (Wissel, 1984). For example, recovery rate was monitored for cyanobacterial cultures exposed to a dilution perturbation by flushing out 10% of the population volume; the recovery rate decreased as the population lost resilience and approached a tipping point where function became irrecoverable (Veraart et al., 2012). In this experiment where the recovery over time was readily observable over a relevant timescale, assessing the system’s resilience is straightforward. Such quantification of recovery time is important because it is known that systems nearing the verge of collapse or those trending toward unstable dynamics frequently exhibit reduced rates of recovery, or decreasing resilience (van Nes and Scheffer, 2007). This is known as “critical slowing down,” meaning that, as a system nears a tipping point, recovery of function after a perturbation slackens (Scheffer et al., 2009, 2012). Thus quantitative measures of resilience are essential for early warning of impending system collapse, from which function cannot be regained.

Although recovery rate provides a direct measurement of resilience, in some systems it is not possible or practical to measure recovery time on time scales relevant to the perturbation. Additionally, some systems do not allow for measurement of resilience because the experiments needed for quantify recovery time are impossible, unethical (e.g., when imposing a perturbation and monitoring function would endanger ecosystems or humans), or otherwise detrimental to the function of neighboring systems. In such cases not amenable to temporal analysis, resilience can alternatively be quantified in terms of *spatial* recovery as a proxy for temporal recovery (Dai et al., 2013). This means that resilience may still be predicted by observing spatial, rather than temporal, features of the system in cases where temporal and spatial recovery are reasonably correlated. The ability to monitor microbial function over relevant spatial scales has become increasingly possible due to advances in imaging and sensor capabilities, enabling a link between function, structure, and microbial identity (Behrens et al., 2008; Li et al., 2008; Pett-Ridge and Weber, 2012; Babauta et al., 2014; Vanwonterghem et al., 2014).

Recently, Dai et al. (2013) put forward recovery length as a measure for resilience. Recovery length is characterized by the distance required for function to recover from a spatial perturbation, and it was initially based on the observation that recovery length, which was correlated to recovery time, increased when a yeast system was on the verge of collapse. In their experiment, populations of *Saccharomyces cerevisiae* were connected spatially along a one-dimensional array through discrete dispersal events. Population stability was measured as the dilution factor was increased, imposing a perturbation. Recovery length was quantified by measuring the population density across distance, which provided a warning signal of imminent population collapse as the dilution factor approached unsustainable levels. Furthermore, these indicators increased with gradual increases in dilution factor, revealing deterioration of resilience in the system in real time. The recovery length concept proposed by Dai and coworkers faithfully quantified resilience to perturbations with sharp boundaries (such as step-change perturbations). As the authors pointed out, however, the same may not be effective for more realistic forms of perturbations that follow gradients or which are blurred across the spatial dimension. In reality, perturbation may come as gradients across both time and space. For example, spatial perturbations that form gradients include the intrusion of substrate or toxins into soil and its subsequent removal (Cao et al., 2012; Moran et al., 2014) or sunlight penetrating into a microbial mat (Bhaya et al., 2001; Häder and Lebert, 2001; Lindemann et al., 2013).

In this study we propose a perturbation-independent resilience metric, termed perturbation-adjusted spatial metric of resilience (PASMORE), to be applicable to perturbations that may be either step-change or gradient-based, and thus extending the recovery length work by Dai et al. (2013). To test this new metric, we developed a simple model of a microbial mat containing species with differing resilience. We discuss cases where the previously proposed metric fails to faithfully measure resilience under gradient perturbations, and then demonstrate how PASMORE provides an appropriate assessment of resilience for the same and other types of perturbation profiles. We also explored the impact of limited knowledge about the shape of perturbation profiles on the effectiveness of PASMORE. Finally, we investigated a test application of PASMORE to systems of two interacting species, and observed how resilience is affected by interspecies competition.

## Model System

**FIGURE 1:**
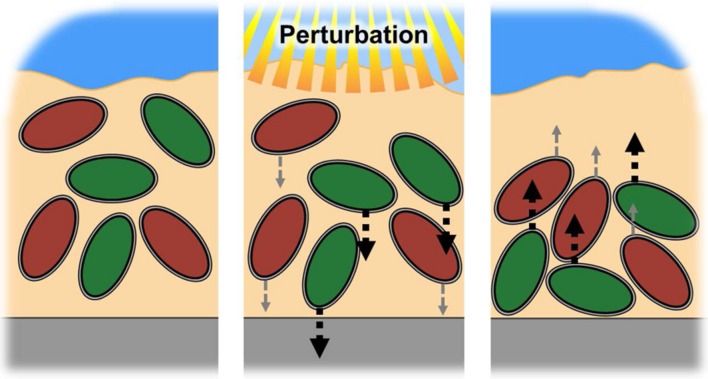
**Schematic of model system.** A microbial mat has a population of species with differing resilience. In this system, resilience is conferred by movement, with rapid movement (green) having higher resilience than slower movement (red). Both cells respond to a perturbation, in this case light penetration into the mat, by moving out of the zone affected by the perturbation. After the perturbation concludes, the cells recover their initial cell density by moving back into the previously affected region. The faster moving cell has a higher resilience, i.e., the ability to recover population density more quickly, as it is capable of returning more quickly to the previously vacated space.

We considered a simple mathematical model, which simulates a microbial mat with populations of microbial species: one with faster motility, and another with slower motility (see **Figure 1** for illustration). Each species responds to a perturbation (e.g., light) with movement proportional and opposite to the perturbation; during perturbation, both species move away from the perturbation toward a region where the perturbation is not present. The key aspect of this simulation is that the motility allows the microbial species to recover their initial population density after the perturbation occurs, with the more motile species recovering more quickly, and thus displaying higher resilience. While resilience is bestowed by motility rate in our model system, there are many other properties of a microbial species that can confer resilience beyond motility, and these features may present themselves within spatial constructions besides microbial mats. Furthermore, our proposed resilience measure has been formulated to be agnostic to the type of function being monitored. As discussed in Song et al. (2015), it is the researcher’s job to identify the function of interest that is measured in a spatially defined community. Function may be defined as any trait or variable of interest, and resilience of the chosen function must be relative to a specific perturbation (Felix and Wagner, 2008). This is the cardinal “What to What?” question discussed by Carpenter et al. (2001) and Lesne (2008). For an in-depth discussion on the biological features that contribute to microbial resilience see the review by Shade et al. (2012). For the sake of evaluating our proposed resilience measure, motility suffices to provide a good working model, with population density as the example function of interest (or as a proxy for community functions that are closely linked to it).

The simulation was built using Comsol Multiphysics (v. 5.0.1.276) finite element analysis software with the chemical reaction engineering module. The microbial mat model was implemented using the diffusion application mode, with a one-dimensional geometry, similar to models previously described by our group (Renslow et al., 2013a,b,c).

## The Concept of the Recovery Length and Proposed Extension

Dai et al. (2013) proposed the half-point recovery length definition of spatial recovery, which increases in proportion to the loss of resilience. The half-point recovery length is defined as *L^half^* in Equation 1:

$$f(L^{half})=\frac{1}{2}[f(x^{b})+f\left(x^{eq}\right)]\;$$

where *f* is the function of interest, *x^b^* is the spatial distance at the boundary of the perturbation, *f(x^eq^)* is the functional value at the corresponding equilibrium (e.g., unperturbed) condition (a diagram of these terms is provided in Dai et al. (2013) supplementary information). For our simulation, we define *f(x^eq^)* as the functional value when it has reached 99% of its true equilibrium value, similar to how we calculate temporal recovery, described below. The equilibrium condition may also be considered to be *f(x^eq^)* where the function is fully perturbed, especially in the case where recovery length is measured at a boundary or where a full recovery profile (i.e., one that includes both perturbed and unperturbed equilibrium regions) may not be available (see Dai et al., Supplementary Figures 6 and 8 for a more detailed discussion on this special case). In cases where the perturbation boundary is not well defined or unknown, such as during a gradient perturbation, we assign *x^b^* to be the spatial distance at the start of the perturbation. This does not alter the recovery length–resilience correlation for step-change perturbations in our model, but simply allows for quantifying *L^half^* in gradient perturbation cases. Also note that, as described in Dai et al., function profiles are normalized by *f(x^eq^)*, and all results are shown on normalized scales for clarity and ease of comparison.

A new measure proposed in this work, PASMORE takes into account the shape of the perturbation, whether it follows a sharp step-change or a gradient. PASMORE is defined as a weighted integral, where the perturbation profile is the weighting function:

$$PASMORE=\int_{s}p(x)f(x)dx$$

where *f* is the function of interest, p is the perturbation profile, and PASMORE is the integral of *pf* over the defined system space, S. In our simulation, the defined system space is the microbial mat where the perturbation has effect.

## Comparison of Recovery Length and Pasmore in Microbial Mats with Non-Interacting Species

**FIGURE 2:**
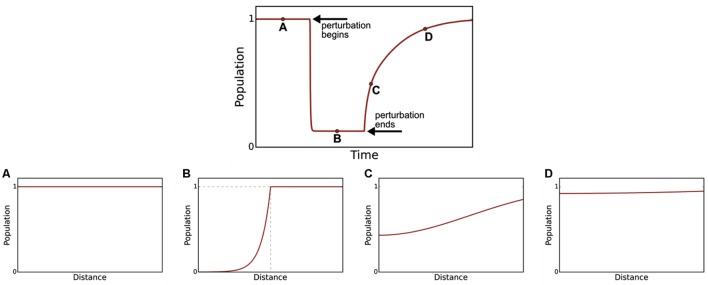
**Recovery over time is associated with recovery through the simulated microbial mat.** The top profile shows the bulk population response of a species over time: before **(A)**, during **(B)**, and after **(C,D)** a step-change perturbation is applied. Bottom figures show the species spatial distribution at each time point. Before the perturbation **(A)** the species has a homogenous population density. During the perturbation **(B)**, with perturbation profile shown as dashed gray line), the population density is lowest in the perturbed region. After the perturbation has been removed, the species begins to recover **(C)** until its population density approaches the pre-perturbed profile **(D)**.

In **Figure 2**, we demonstrate how a microbial population responds to the application and removal of a perturbation. It is possible to see the relationship between temporal recovery and spatial recovery. Temporal recovery is defined as the time required for the recovery of function, in this case population density, to 99% of its original value. In cases where spatial recovery is correlated to temporal recovery, such as in our simulation (Pearson product-moment correlation coefficient, i.e., Pearson’s *r*, of 1.0), spatial recovery metrics may be used as a proxy for quantifying resilience.

**FIGURE 3:**
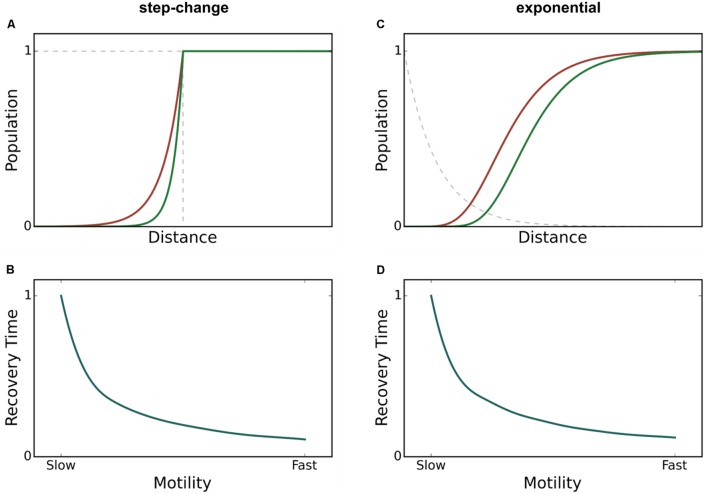
**Spatial population density profiles during a perturbation and recovery time – motility relationship for two different perturbation profiles: step-change **(A,B)** and exponential **(C,D)**.** The faster species (green) recovers more quickly after a perturbation than the slow species (red). Regardless of the shape of the perturbation profile, the recovery time decreases as motility increases, verifying that resilience is higher for the faster species.

**FIGURE 4:**
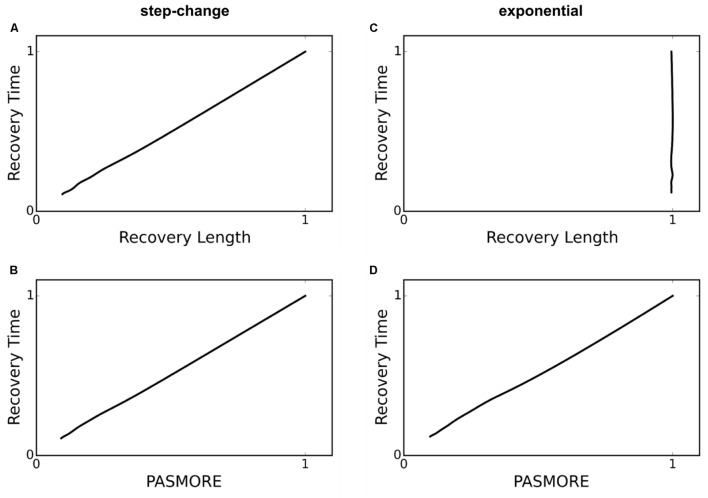
**Recovery time – recovery length and recovery time – PASMORE relationships for two different perturbation profiles: step-change **(A,B)** and exponential **(C,D)**.** For a step-change perturbation, recovery length correctly correlates with recovery time **(A)**, however, it is unable to correctly correlate when the perturbation profile is exponential **(C)**. PASMORE correctly correlates to recovery time regardless of the perturbation profile **(B,D)**.

It is known that perturbations, whether occurring over time or over spatial domains, will occur frequently across gradients of varying sharpness. For example, using the example of light as the source of perturbation, attenuation follows an exponential decay profile. Even when the perturbation occurs across a gradient, rapidly motile species exhibit high resilience, meaning in this case that they recover population density more quickly than species with slow motility. However, as discussed by Dai et al. (2013), half-point recovery length may fail to accurately quantify resilience under conditions of a blurred or gradient perturbation. We tested step-change, linear, polynomial, and exponential perturbation gradients (data shown only for the exponential case) (**Figure 3**). The ability for spatial recovery to correlate to temporal recovery decreases as the perturbation profiles approaches an exponential function, where all correlation is lost (Pearson’s *r* of 0.0). **Figure 4** demonstrates that PASMORE is able to accurately quantify spatial recovery and maintain the correlation to temporal recovery (Pearson’s *r* of 1.0) regardless of the perturbation profile. This was true for all perturbation gradients that we tested. Resilience arises due to the motility of the population, thus the metric should maintain its relationship to motility regardless of the perturbation to which it is exposed.

## Quantifying Resilience with Limited Perturbation Information

**FIGURE 5:**
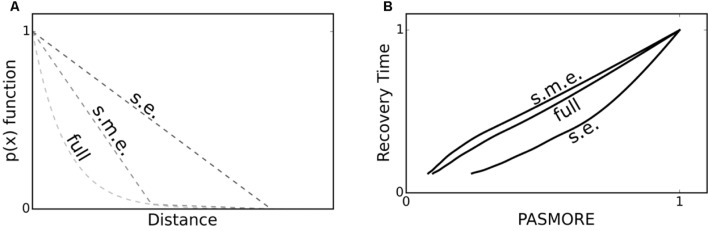
**In some cases, a full perturbation profile, i.e., *p(x)*, may not be known.** However, it may be possible to make a few measurements and interpolate the perturbation profile, for example at the start and end of the perturbation profile. **(A)** Three demonstration cases for a measured perturbation profile: Start and end (s.e., linear approximation), start-middle-end (s.m.e., two linear lines approximation), and full profile (full, exponential). **(B)** Recovery Time – PASMORE correlation to the three measured perturbation profiles. Even in the cases where only limited information about the true perturbation profile is known, PASMORE may still correlate well with recovery time.

Properly quantifying resilience using spatial metrics requires information about the perturbation’s shape. This is true whether PASMORE or other metrics like half-point recovery length are used. However, in experimental systems, it may not be possible to obtain the entire profile of a perturbation gradient. Here we wanted to quantify the effect of limited information about the gradient. Therefore, we examined PASMORE for use when the exact shape of the gradient imposed by a perturbation was uncertain. As shown in **Figure 5**, we compared two cases against the full-profile PASMORE: (1) a two-point dataset to generate a linear approximation between the start and end of the observed perturbation and (2) a three-point dataset to generate two linear approximations between the start and middle and between the middle and end of the observed perturbation. Note that only the perturbation profile was approximated, not the population density, since it is assumed that the function of interest, for which resilience is to be evaluated, is quantifiable. Using sensitive measurement instruments in real systems, it is likely that more than two or three data points of the perturbation profile could be obtained, for example by using microelectrodes (Nguyen et al., 2012) or NMR imaging (Renslow et al., 2010). However, as will be demonstrated, even using a three-point approximation, the calculation of PASMORE quickly approaches the full-profile PASMORE. **Figure 5** shows that PASMORE is able to maintain the correlation to temporal recovery with a two-point approximation (Pearson’s *r* of 0.96), and this correlation improved with a three-point dataset (Pearson’s *r* of 0.99).

## Analysis of a Microbial Mat with Interacting Species

**FIGURE 6:**
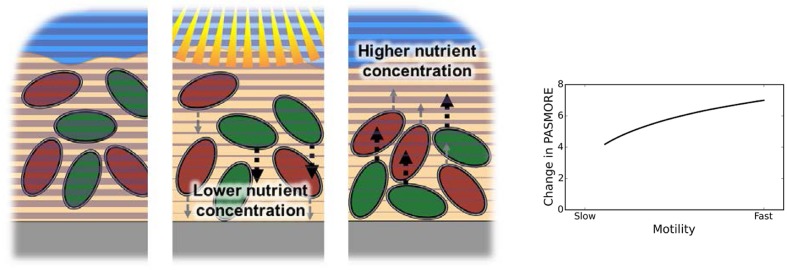
**Schematic of the model system, which has been modified to force competition between two species (one with higher resilience and one with lower resilience) by making motility dependent on consumption of a sole nutrient, which is supplied in the liquid above the microbial mat.** During a perturbation, the nutrient concentration far from the perturbation becomes limited. The right plot shows the percent change of PASMORE when a slower species competes for the nutrient with a faster species, compared to a non-interacting case (i.e., when the species rely on differing nutrients). The competitive interaction led an increase in PASMORE, i.e., lower resilience, for both the slow and fast species due to the competition between species. The faster species exhibited a greater decrease in resilience compared to the slower species.

We investigated how resilience as measured by PASMORE would change if two species in the mat interacted with each other. Therefore, we modified the model to simultaneously include two motile species that exhibited a difference in their rate of motility, but which was dependent on the consumption of a substrate. The substrate diffused from the top of the mat and consumption followed Monod-type kinetics, based on the diffusion and reaction parameters and setup of a previous model (Renslow et al., 2013b). Such a configuration broadly approximates the structure of a cyanobacterial mat, where the carbon fixation upon which heterotrophs depend is maximal near the mat surface (Lindemann et al., 2013). The case where both species competed for the same substrate was compared to the case where the species consumed two independent substrates noncompetitively. **Figure 6** shows the percent change of PASMORE comparing the non-interacting to competitive interaction cases. Compared to the non-interacting case, the interacting species displayed an increase in PASMORE, and thus were found to have lower resilience. Furthermore, the species with the faster motility exhibited a larger loss of resilience compared to the slower species. This is due to the fact that, during perturbation, the species with higher resilience occupy a lower nutrient zone, while the species with lower resilience are closer to the nutrient source and are thus less affected. As such, the model illustrates a trade-off between resource availability and stress avoidance in these motile species; we anticipate that similar trade-offs will exist in many spatially organized communities where local interactions lead to gains in fitness. Finally, this simple simulation demonstrates how PASMORE can help quantify resilience in a case where previous metrics would not have been meaningful.

## Pasmore in the Broader Context of Spatial Resilience Measures

The capability to monitor spatial patterns of microbial function can help elucidate the resilience of a community in cases where temporal measurements may not have previously been practical. Spatial measures remove the need to monitor a given function over time, and thus a snapshot of the present community stability can be gauged. This type of analysis may have implications beyond the microbial world, as there are many forms of complex networks and communities that display properties of emergent phenomena with spatially-relevant functions. For example, the spatial effects of social dilemmas have been investigated for several decades (Nowak and May, 1992; Hauert, 2006) and the frequency of human cooperation and collaboration or selfishness and exploitation across spatial dimensions impacts the resilience of groups of peoples and their ability to maintain high productivity (Alvard, 2004; Jimenez et al., 2008). Furthermore, spatial patterns of human settlements and population densities may be related to community stability in the face of perturbation events such as loss of water, tillable soil, hunting ground, food, energy and other resources, as well as the related incidents of overpopulation and politico-military conflicts (Tir and Diehl, 1998; Vandam et al., 2013). In this context, densities of refugee settlements across countries and subsequent patterns of repopulation of cities after wars may reveal human community and ethno-regional group stability properties in the face of significant devastation. Other possible application areas for spatial measures of resilience could include aquifer-groundwater recharge rates (Guglielmi and Mudry, 1996; Katic and Grafton, 2011), soil and vegetation health (Seybold et al., 1999; van de Koppel and Rietkerk, 2004; Alongi, 2008; Jiang et al., 2012), permafrost vulnerability (Jorgenson et al., 2010), coral reef habitats (Nystrom and Folke, 2001; Cheal et al., 2013), and fishery management (Carpenter and Brock, 2004; Kerr et al., 2010). Indeed, understanding the resilience of biological and non-biological systems will be critical for determining the impact of climate change and the necessary policy changes to alleviate negative consequences (Bloetscher et al., 2010; Cumming, 2011). Future experiments and observations will need to be done to determine the extent to which spatial measures of resilience, such as PASMORE, may be used to provide insight into stability of complex systems beyond microbial communities. Furthermore, the ability of PASMORE to measure resilience in highly heterogeneous, discontinuous, or physically-restricted systems will need to be tested. It is unclear how barriers that constrain functional recovery, whether social, cultural, financial, political, or physical (e.g., soil and minerals at the microbial scale), will impact the capacity of PASMORE to measure resilience.

## Conclusion

‘Perturbation-adjusted spatial metric of resilience’ (PASMORE) is capable of accurately quantifying spatial recovery and maintaining the correlation to temporal recovery regardless of the perturbation profile. In many environmental and ecological systems, the gradient shape of the perturbation may not be quantifiable and even the identity or type of perturbation may not be known. However, even with limited information, PASMORE can be calculated with approximation of the perturbation profile to reveal a close estimate of the system’s resilience. We envision that establishing the concept of PASMORE as a practically useful metric of resilience requires further studies, including investigation of the limits of using spatial recovery in lieu of temporal recovery to quantify resilience in real natural and engineered microbial communities and relating these measurements to predictions of when systems are on the verge of collapse or nearing an irreversible transition across a tipping point. The present work provides initial foundations along this direction.

## Author Contributions

All authors listed have made substantial, direct, and intellectual contribution to the work, and approved it for publication.

## Conflict of Interest Statement

The authors declare that the research was conducted in the absence of any commercial or financial relationships that could be construed as a potential conflict of interest.

## References

[B1] Allison S. D. Martiny J. B. H. (2008). Resistance, resilience, and redundancy in microbial communities. *Proc. Natl. Acad. Sci. U.S.A.* 105 11512–11519. 10.1073/pnas.0801925105 18695234 PMC2556421

[B2] Alongi D. M. (2008). Mangrove forests: resilience, protection from tsunamis, and responses to global climate change. *Estuar. Coast. Shelf Sci.* 76 1–13. 10.1016/j.ecss.2007.08.024

[B3] Alvard M. (2004). Good hunters keep smaller shares of larger pies. *Behav. Brain Sci.* 27 560–561. 10.1017/S0140525X0422012X

[B4] Babauta J. T. Atci E. Ha P. T. Lindemann S. R. Ewing T. Call D. R. (2014). Localized electron transfer rates and microelectrode-based enrichment of microbial communities within a phototrophic microbial mat. *Front. Microbiol.* 5:11 10.3389/fmicb.2014.00011 PMC3902354 24478768

[B5] Behrens S. Losekann T. Pett-Ridge J. Weber P. K. Ng W. O. Stevenson B. S. (2008). Linking microbial phylogeny to metabolic activity at the single-cell level by using enhanced element labeling-catalyzed reporter deposition fluorescence in situ hybridization (EL-FISH) and NanoSIMS. *Appl. Environ. Microbiol.* 74 3143–3150. 10.1128/AEM.00191-08 18359832 PMC2394947

[B6] Bernstein H. C. Beam J. P. Kozubal M. A. Carlson R. P. Inskeep W. P. (2013). In situ analysis of oxygen consumption and diffusive transport in high-temperature acidic iron-oxide microbial mats. *Environ. Microbiol.* 15 2360–2370. 10.1111/1462-2920.12109 23516993

[B7] Bernstein H. C. Kesaano M. Moll K. Smith T. Gerlach R. Carlson R. P. (2014). Direct measurement and characterization of active photosynthesis zones inside wastewater remediating and biofuel producing microalgal biofilms. *Bioresour. Technol.* 156 206–215. 10.1016/j.biortech.2014.01.001 24508901

[B8] Bhaya D. Takahashi A. Grossman A. R. (2001). Light regulation of type IV pilus-dependent motility by chemosensor-like elements in *Synechocystis* PCC6803. *Proc. Natl. Acad. Sci. U.S.A.* 98 7540–7545. 10.1073/pnas.131201098 11404477 PMC34704

[B9] Bloetscher F. Meeroff D. E. Heimlich B. N. Brown A. R. Bayler D. Loucraft M. (2010). Improving resilience against the effects of climate change. *J. Am. Water Works Assoc.* 102 36–46. 10.1007/s00572-014-0595-2

[B10] Cao B. Ahmed B. Kennedy D. W. Wang Z. Shi L. Marshall M. J. (2011). Contribution of extracellular polymeric substances from *Shewanella* sp. HRCR-1 biofilms to U (VI) immobilization. *Environ. Sci. Technol.* 45 5483–5490. 10.1021/es200095j 21627155

[B11] Cao B. Majors P. D. Ahmed B. Renslow R. S. Silvia C. P. Shi L. (2012). Biofilm shows spatially stratified metabolic responses to contaminant exposure. *Environ. Microbiol.* 14 2901–2910. 10.1111/j.1462-2920.2012.02850.x 22925136 PMC3480979

[B12] Carey M. Carville S. (2003). Scheduling and platforming trains at busy complex stations. *Transp. Res. Part A Policy Pract.* 37 195–224. 10.1016/S0965-8564(02)00012-5

[B13] Carpenter S. Walker B. Anderies J. M. Abel N. (2001). From metaphor to measurement: resilience of what to what? *Ecosystems* 4 765–781. 10.1007/s10021-001-0045-9

[B14] Carpenter S. R. Brock W. A. (2004). Spatial complexity, resilience, and policy diversity: fishing on lake-rich landscapes. *Ecol. Soc.* 9 31.

[B15] Cheal A. J. Emslie M. Macneil M. A. Miller I. Sweatman H. (2013). Spatial variation in the functional characteristics of herbivorous fish communities and the resilience of coral reefs. *Ecol. Appl.* 23 174–188. 10.1890/11-2253.1 23495645

[B16] Cimellaro G. P. Reinhorn A. M. Bruneau M. (2010). Seismic resilience of a hospital system. *Struct. Infrastruct. Eng.* 6 127–144. 10.1080/15732470802663847

[B17] Cumming G. S. (2011). Spatial resilience: integrating landscape ecology, resilience, and sustainability. *Landsc. Ecol.* 26 899–909. 10.1007/s10980-011-9623-1

[B18] Curtis T. P. Sloan W. T. (2004). Prokaryotic diversity and its limits: microbial community structure in nature and implications for microbial ecology. *Curr. Opin. Microbiol.* 7 221–226. 10.1016/j.mib.2004.04.010 15196488

[B19] Czaczyk K. Myszka K. (2007). Biosynthesis of extracellular polymeric substances (EPS) and its role in microbial biofilm formation. *Pol. J. Environ. Stud.* 16 799–806.

[B20] Dai L. Korolev K. S. Gore J. (2013). Slower recovery in space before collapse of connected populations. *Nature* 496 355–358. 10.1038/nature12071 23575630 PMC4303252

[B21] De La Fuente I. M. Martínez L. Pérez-Samartín A. L. Ormaetxea L. Amezaga C. Vera-López A. (2008). Global self-organization of the cellular metabolic structure. *PLoS ONE* 3:e3100 10.1371/journal.pone.0003100 PMC2519785 18769681

[B22] Easterling W. E. Kok K. (2003). “Emergent properties of scale in global environmental modeling: are there any?,” in *Scaling in Integrated Assessment*, eds Rotmans J. Rothman D. S. (Lisse: Swets and Zeitlinger Group BV), 263–292.

[B23] Felix M. A. Wagner A. (2008). Robustness and evolution: concepts, insights and challenges from a developmental model system. *Heredity* 100 132–140. 10.1038/sj.hdy.6800915 17167519

[B24] Flemming H. C. Wingender J. (2001). Relevance of microbial extracellular polymeric substances (EPSs) – Part I: structural and ecological aspects. *Water Sci. Technol.* 43 1–8. 11381954

[B25] Fuhrman J. A. (2009). Microbial community structure and its functional implications. *Nature* 459 193–199. 10.1038/nature08058 19444205

[B26] Griffiths B. S. Philippot L. (2013). Insights into the resistance and resilience of the soil microbial community. *FEMS Microbiol. Rev.* 37 112–129. 10.1111/j.1574-6976.2012.00343.x 22568555

[B27] Guglielmi Y. Mudry J. (1996). Estimation of spatial and temporal variability of recharge fluxes to an alluvial aquifer in a fore land area by water chemistry and isotopes. *Ground Water* 34 1017–1023. 10.1111/j.1745-6584.1996.tb02167.x

[B28] Häder D.-P. Lebert M. (2001). *Photomovement.* Amsterdam: Elsevier Science.

[B29] Hauert C. (2006). Spatial effects in social dilemmas. *J. Theor. Biol.* 240 627–636. 10.1016/j.jtbi.2005.10.024 16352316

[B30] Hawkes C. V. Keitt T. H. (2015). Resilience vs. historical contingency in microbial responses to environmental change. *Ecol. Lett.* 18 612–625. 10.1111/ele.12451 25950733

[B31] Hibiya K. Terada A. Tsuneda S. Hirata A. (2003). Simultaneous nitrification and denitrification by controlling vertical and horizontal microenvironment in a membrane-aerated biofilm reactor. *J. Biotechnol.* 100 23–32. 10.1016/S0168-1656(02)00227-4 12413783

[B32] Higgins P. A. T. Mastrandrea M. D. Schneider S. H. (2002). Dynamics of climate and ecosystem coupling: abrupt changes and multiple equilibria. *Philos. Trans. R. Soc. Lond. B Biol. Sci.* 357 647–655. 10.1098/rstb.2001.1043 12079526 PMC1692972

[B33] Jiang J. Gao D. Z. Deangelis D. L. (2012). Towards a theory of ecotone resilience: coastal vegetation on a salinity gradient. *Theor. Popul. Biol.* 82 29–37. 10.1016/j.tpb.2012.02.007 22441163

[B34] Jimenez R. Lugo H. Cuesta J. A. Sanchez A. (2008). Emergence and resilience of cooperation in the spatial prisoner’s dilemma via a reward mechanism. *J. Theor. Biol.* 250 475–483. 10.1016/j.jtbi.2007.10.010 18045621

[B35] Jorgenson M. T. Romanovsky V. Harden J. Shur Y. O’donnell J. Schuur E. A. G. (2010). Resilience and vulnerability of permafrost to climate change. *Can. J. For. Res.* 40 1219–1236. 10.1139/X10-060

[B36] Katic P. Grafton R. Q. (2011). Optimal groundwater extraction under uncertainty: resilience versus economic payoffs. *J. Hydrol.* 406 215–224. 10.1016/j.jhydrol.2011.06.016

[B37] Kerr L. A. Cadrin S. X. Secor D. H. (2010). The role of spatial dynamics in the stability, resilience, and productivity of an estuarine fish population. *Ecol. Appl.* 20 497–507. 10.1890/08-1382.1 20405802

[B38] Konopka A. (2009). What is microbial community ecology? *ISME J.* 3 1223–1230. 10.1038/ismej.2009.88 19657372

[B39] Laspidou C. S. Rittmann B. E. (2002). A unified theory for extracellular polymeric substances, soluble microbial products, and active and inert biomass. *Water Res.* 36 2711–2720. 10.1016/S0043-1354(01)00414-6 12146858

[B40] Lesne A. (2008). Robustness: confronting lessons from physics and biology. *Biol. Rev.* 83 509–532. 10.1111/j.1469-185X.2008.00052.x 18823391

[B41] Li T. Wu T. D. Mazeas L. Toffin L. Guerquin-Kern J. L. Leblon G. (2008). Simultaneous analysis of microbial identity and function using NanoSIMS. *Environ. Microbiol.* 10 580–588. 10.1111/j.1462-2920.2007.01478.x 18028417 PMC2253709

[B42] Lindemann S. R. Moran J. J. Stegen J. C. Renslow R. S. Hutchison J. R. Cole J. K. (2013). The epsomitic phototrophic microbial mat of Hot Lake, Washington: community structural responses to seasonal cycling. *Front. Microbiol.* 4:323 10.3389/fmicb.2013.00323 PMC3826063 24312082

[B43] Lusseau D. (2003). The emergent properties of a dolphin social network. *Proc. R. Soc. B Biol. Sci.* 270 S186–S188. 10.1098/rsbl.2003.0057 PMC1809954 14667378

[B44] Mitrovic M. Tadic B. (2010). Bloggers behavior and emergent communities in Blog space. *Eur. Phys. J. B* 73 293–301. 10.1140/epjb/e2009-00431-9

[B45] Moran J. J. Doll C. G. Bernstein H. C. Renslow R. S. Cory A. B. Hutchison J. R. (2014). Spatially tracking C-13-labelled substrate (bicarbonate) accumulation in microbial communities using laser ablation isotope ratio mass spectrometry. *Environ. Microbiol. Rep.* 6 786–791. 10.1111/1758-2229.12211 25155264

[B46] Nguyen H. D. Renslow R. Babauta J. Ahmed B. Beyenal H. (2012). A voltammetric flavin microelectrode for use in biofilms. *Sens. Actuators B Chem.* 161 929–937. 10.1016/j.snb.2011.11.066 22368323 PMC3285096

[B47] Nowak M. A. May R. M. (1992). Evolutionary games and spatial chaos. *Nature* 359 826–829. 10.1038/359826a0

[B48] Nystrom M. Folke C. (2001). Spatial resilience of coral reefs. *Ecosystems* 4 406–417. 10.1007/s10021-001-0019-y

[B49] Pett-Ridge J. Weber P. K. (2012). “NanoSIP: nanoSIMS applications for microbiology,” in *Springer Protocols*, ed. Navid A. (New York, NY: Humana Press), 375–408.

[B50] Porter M. A. Mucha P. J. Newman M. E. J. Warmbrand C. M. (2005). A network analysis of committees in the US House of Representatives. *Proc. Natl. Acad. Sci. U.S.A.* 102 7057–7062. 10.1073/pnas.0500191102 15897470 PMC1129104

[B51] Renslow R. Babauta J. Kuprat A. Schenk J. Ivory C. Fredrickson J. (2013a). Modeling biofilms with dual extracellular electron transfer mechanisms. *Phys. Chem. Chem. Phys.* 15 19262–19283. 10.1039/c3cp53759e 24113651 PMC3868370

[B52] Renslow R. Lewandowski Z. Beyenal H. (2011). Biofilm image reconstruction for assessing structural parameters. *Biotechnol. Bioeng.* 108 1383–1394. 10.1002/bit.23060 21280029 PMC3076525

[B53] Renslow R. S. Babauta J. T. Dohnalkova A. C. Boyanov M. I. Kemner K. M. Majors P. D. (2013b). Metabolic spatial variability in electrode-respiring Geobacter sulfurreducens biofilms. *Energy Environ. Sci.* 6 1827–1836. 10.1039/c3ee40203g 23930138 PMC3733395

[B54] Renslow R. S. Babauta J. T. Majors P. D. Beyenal H. (2013c). Diffusion in biofilms respiring on electrodes. *Energy Environ. Sci.* 6 595–607. 10.1039/C2EE23394K 23420623 PMC3571104

[B55] Renslow R. S. Majors P. D. Mclean J. S. Fredrickson J. K. Ahmed B. Beyenal H. (2010). In situ effective diffusion coefficient profiles in live biofilms using pulsed-field gradient nuclear magnetic resonance. *Biotechnol. Bioeng.* 106 928–937. 10.1002/bit.22755 20589671 PMC2898744

[B56] Robinson C. J. Bohannan B. J. M. Young V. B. (2010). From structure to function: the ecology of host-associated microbial communities. *Microbiol. Mol. Biol. Rev.* 74 453–476. 10.1128/MMBR.00014-10 20805407 PMC2937523

[B57] Scheffer M. Bascompte J. Brock W. A. Brovkin V. Carpenter S. R. Dakos V. (2009). Early-warning signals for critical transitions. *Nature* 461 53–59. 10.1038/nature08227 19727193

[B58] Scheffer M. Carpenter S. R. Lenton T. M. Bascompte J. Brock W. Dakos V. (2012). Anticipating critical transitions. *Science* 338 344–348. 10.1126/science.1225244 23087241

[B59] Schramm A. Larsen L. H. Revsbech N. P. Ramsing N. B. Amann R. Schleifer K. H. (1996). Structure and function of a nitrifying biofilm as determined by in situ hybridization and the use of microelectrodes. *Appl. Environ. Microbiol.* 62 4641–4647. 8953735 10.1128/aem.62.12.4641-4647.1996 PMC168290

[B60] Schubert W. (2014). Systematic, spatial imaging of large multimolecular assemblies and the emerging principles of supramolecular order in biological systems. *J. Mol. Recognit.* 27 3–18. 10.1002/jmr.2326 24375580 PMC4283051

[B61] Seybold C. A. Herrick J. E. Brejda J. J. (1999). Soil resilience: a fundamental component of soil quality. *Soil Sci.* 164 224–234. 10.1097/00010694-199904000-00002

[B62] Shade A. Peter H. Allison S. D. Baho D. L. Berga M. Burgmann H. (2012). Fundamentals of microbial community resistance and resilience. *Front. Microbiol.* 3:417 10.3389/fmicb.2012.00417 PMC3525951 23267351

[B63] Song H.-S. Renslow R. S. Fredrickson J. K. Lindemann S. R. (2015). Integrating ecological and engineering concepts of resilience in microbial communities. *Front. Microbiol.* 6:1298 10.3389/fmicb.2015.01298 PMC4664643 26648912

[B64] Tilman D. Reich P. B. Knops J. Wedin D. Mielke T. Lehman C. (2001). Diversity and productivity in a long-term grassland experiment. *Science* 294 843–845. 10.1126/science.1060391 11679667

[B65] Tir J. Diehl P. F. (1998). Demographic pressure and interstate conflict: linking population growth and density to militarized disputes and wars, 1930-89. *J. Peace Res.* 35 319–339. 10.1177/0022343398035003004

[B66] van de Koppel J. Rietkerk M. (2004). Spatial interactions and resilience in arid ecosystems. *Am. Nat.* 163 113–121. 10.1086/380571 14767841

[B67] van Nes E. H. Scheffer M. (2007). Slow recovery from perturbations as a generic indicator of a nearby catastrophic shift. *Am. Nat.* 169 738–747. 10.1086/516845 17479460

[B68] Vandam R. Kaptijn E. Vanschoenwinkel B. (2013). Disentangling the spatio-environmental drivers of human settlement: an eigenvector based variation decomposition. *PLoS ONE* 8:e67726 10.1371/journal.pone.0067726 PMC3699633 23844076

[B69] Vanwonterghem I. Jensen P. D. Ho D. P. Batstone D. J. Tyson G. W. (2014). Linking microbial community structure, interactions and function in anaerobic digesters using new molecular techniques. *Curr. Opin. Biotechnol.* 27 55–64. 10.1016/j.copbio.2013.11.004 24863897

[B70] Veraart A. J. Faassen E. J. Dakos V. Van Nes E. H. Lurling M. Scheffer M. (2012). Recovery rates reflect distance to a tipping point in a living system. *Nature* 481 357–359. 10.1038/nature10723 22198671

[B71] Wagner M. Nielsen P. H. Loy A. Nielsen J. L. Daims H. (2006). Linking microbial community structure with function: fluorescence in situ hybridization-microautoradiography and isotope arrays. *Curr. Opin. Biotechnol.* 17 83–91. 10.1016/j.copbio.2005.12.006 16377170

[B72] Wintermute E. H. Silver P. A. (2010). Emergent cooperation in microbial metabolism. *Mol. Syst. Biol.* 6 407 10.1038/msb.2010.66 PMC2964121 20823845

[B73] Wissel C. (1984). A universal law of the characteristic return time near thresholds. *Oecologia* 65 101–107. 10.1007/BF00384470 28312117

[B74] Xavier J. B. Foster K. R. (2007). Cooperation and conflict in microbial biofilms. *Proc. Natl. Acad. Sci. U.S.A.* 104 876–881. 10.1073/pnas.0607651104 17210916 PMC1783407

